# Physiological and Transcriptomic Analyses Elucidate That Exogenous Calcium Can Relieve Injuries to Potato Plants (*Solanum tuberosum* L.) under Weak Light

**DOI:** 10.3390/ijms20205133

**Published:** 2019-10-16

**Authors:** Jinfeng Hou, Jie Li, Yang Yang, Zixin Wang, Bowen Chang, Xiaowei Yu, Lingyun Yuan, Chenggang Wang, Guohu Chen, Xiaoyan Tang, Shidong Zhu

**Affiliations:** 1 College of Horticulture, Vegetable Genetics and Breeding Laboratory, Anhui Agricultural University, Hefei 230036, China; houjinfeng@ahau.edu.cn (J.H.); jieli@ahau.edu.cn (J.L.); yangyang1028@ahau.edu.cn (Y.Y.); 18721085@ahau.edu.cn (Z.W.); changbowen999@163.com (B.C.); yxw19681109@163.com (X.Y.); ylyun99@163.com (L.Y.); cgwang@ahau.edu.cn (C.W.); cgh@ahau.edu.cn (G.C.); yitangxy@126.com (X.T.); 2 Provincial Engineering Laboratory for Horticultural Crop Breeding of Anhui, Hefei 230036, China; 3 Department of Vegetable Culture and Breeding, Wanjiang Vegetable Industrial Technology Institute, Maanshan 238200, China

**Keywords:** Ca^2+^, potato, weak light, transcriptome, relieve injuries, photosynthesis

## Abstract

Light is one of the most important abiotic factors for most plants, which affects almost all growth and development stages. In this study, physiological indicators suggest that the application of exogenous Ca^2+^ improves photosynthesis and changes phytohormone levels. Under weak light, photosynthetic parameters of the net photosynthetic rate (P*_N_*), stomatal conductance (Gs), and transpiration rate (Tr) decreased; the antioxidation systems peroxidase (POD), superoxide dismutase (SOD), and catalase (CAT) reduced; the degrees of malondialdehyde (MDA), H_2_O_2_, and superoxide anion (O_2_^−^) free radical damage increased; while exogenous Ca^2+^ treatment was significantly improved. RNA-seq analysis indicated that a total of 13,640 differently expressed genes (DEGs) were identified and 97 key DEGs related to hormone, photosynthesis, and calcium regulation were differently transcribed. Gene ontology (GO) terms and Kyoto encyclopedia of genes and genomes (KEGG) pathway analyses, plant hormone signal transduction, photosynthesis, carbon metabolism, and phenylpropanoid biosynthesis were significantly enriched. Additionally, quantitative real-time PCR (qRT-PCR) analysis confirmed some of the key gene functions in response to Ca^2+^. Overall, these results provide novel insights into the complexity of Ca^2+^ to relieve injuries under weak light, and they are helpful for potato cultivation under weak light stress.

## 1. Introduction

Potatoes (*Solanum tuberosum* L.) are an important food and vegetable crop grown throughout the world, and they are a high-yield crop with good prospects for development. In the early stages of potato growth, plant growth is easily impaired, which not only inhibits photosynthesis but also ultimately reduces yield and quality under weak light stress [1]. Light is a significant factor that affects plant growth, and the change in light intensity has a great impact on plant growth, development, and photosynthesis, including various aspects of plant physiology and cell biochemistry [2]. Shade inhibits light absorption, thereby reducing the photosynthetic ability and limiting the growth of plants. Under weak light stress, plants usually reduce branches, lengthen internodes, thin stems, increase leaf area, increase stem length, and decrease internodes [3]. In weak light, in order to ensure the normal progress of photosynthesis, plants usually give priority to supplying their accumulated products to leaves, which leads to the thinning of leaves [4]. These phenotypic variations usually lead to lodging and the plants being more susceptible to disease. While weak light stress is very common in potato cultivation in the middle and lower reaches of the Yangtze river in China (e.g., steadily rainy weather in early spring), protected cultivation and interplanting cultivation usually accompany the weak light environment.

Calcium (Ca^2+^) is an important component of the plant cell wall and cell membrane structure, and it is an essential nutrient for plant growth and development. Ca^2+^ is a secondary messenger in plant cells. Its expression level changes in response to the plant environment and endogenous hormones (such as plant hormones, stress, diseases, etc.), which will change the level of intracellular Ca^2+^ [5]. Studies have shown that extracellular Ca^2+^, as extracellular messengers, can coordinate changes in intracellular Ca^2+^, thus determining the role of Ca^2+^ as a messenger in plants [6]. Ca^2+^ helps protect the stability of cell membranes, cell walls, and membrane-bound proteins, and it plays a crucial role in regulating the transport of inorganic ions in plant cells [7]. Ca^2+^ regulation plays a role throughout the entire process of plant germination, growth and differentiation, morphogenesis, and flowering [8]. Calcium-binding protein (CaM), which has a strong affinity for Ca^2+^, is commonly found in plant cells. When the plant is stimulated by the outside to a certain extent, Ca^2+^ binding to CaM causes a change in the CaM conformation. CaM is activated and binds to the target enzyme, and the target enzyme is activated by a Ca^2+^–CaM-dependent protein kinase, resulting in a series of physiological and biochemical reactions [9]. Plant extracellular Ca^2+^ participates in the formation of the cell wall structure, and it maintains the structure and function of the cell membrane by lining the membrane with phospholipids and proteins that affect the outer surface of the plasma membrane. When intracellular Ca^2+^ is insufficient, it can provide an extracellular Ca^2+^ source and participate in regulation [10]. 

Most current research suggests that the shade avoidance response of plants is mainly affected by phytochrome (PHY) regulation [11]. Studies have revealed that in *Arabidopsis* plants, shade avoidance is the main response when light intensity is not sufficient. This is caused by the ability of the PHY photoreceptor to perceive a reduction in red/far-red (R/FR) ratio [12], with its basic spiral–ring–spiral (bHLH) protein family interaction, namely the phytochrome interaction factor (PIF) interactions [13]. Meanwhile, the DELLA protein (triggers the degradation of plant growth repressor DELLA proteins) stability decreases, the binding force with PIFs decreases [14], and the activity and accumulation of PIFs increase, so as to promote the expression of downstream genes related to shade avoidance responses and promote plant growth. Among them, gibberellin (GA), ethylene, and auxin are also involved in the shading reaction, including hypocotyl and petiole extensions [15,16]. Plant hormones have different effects on plant growth and development [17], and can be used to reduce the negative effects of shadows.

Plant endogenous hormones such as auxin, gibberellin, ethylene, and cytokinin are involved in plant responses to shading conditions. Studies have shown that the synthesis of auxin (IAA) with the participation of photosensitive pigments [18], transport, and recognition [19] are all related to plant shade avoidance responses. GA can release PIFs by binding to DELLAs, and the DELLAs bound to GA are degraded by the 26S proteasome; thus, PIFs are prevented from binding to the DELLAs proteins, which promotes plant growth and the shading avoidance reaction [20]. Ethylene is an important hormone that regulates the internode elongation of plants, and it has been shown to be involved in shade avoidance reactions of various plants. The ethylene content will increase in transgenic plants overexpressing PIF5 [21]. Photochromes, cryptochromes, and GA also interact with each other in pairs or in multiple groups to regulate the elongation of hypocotyls or internodes. Changes to the auxin response in gene expression is an important link in the auxin signal transduction pathway. SAUR (Small Auxin Up RNA) is the largest gene family and responds early to auxin. Auxin has long been known to induce rapid changes in Ca^2+^ concentration in plant cells. Bioinformatics analysis results show that most SAUR is divided between family proteins in *Arabidopsis* and may be combined with the CaM site [22].

Plants carry out material conversion and energy metabolism through photosynthesis, and their physiological processes are very sensitive to weak light stress. Under weak light stress, plants must absorb and capture as much light energy as possible so as to accelerate the fixation of CO_2_ and the accumulation of carbohydrates for the normal growth and development of plants. Plants under weak light stress change their light compensation point, light saturation point, photosynthetic enzyme activity, photosynthetic site structure, and electron transfer. Studies have shown that, with the enhancement of weak light stress and the decrease of effective photosynthetic radiation, the light compensation point, light saturation point, maximum net photosynthetic rate, and dark respiration rate of plants all show decreasing trends, and the chlorophyll (Chl) content in leaves increased, while Chl a/b decreased [23]. Studies have shown that the changes in stomatal conductance and intercellular CO_2_ concentration of cotton leaves under shading conditions were not the reasons for the decreased net photosynthetic rate. The photosynthetic electron transfer capacity of cotton leaves under shading conditions determines the net photosynthetic rate [24]. Fay and Knapp found a certain correlation between a lag in the photosynthetic rate and stomatal conductance through short-term light responses in soybean leaves [25]. Plant cells in a normal environment have a normal activity with their enzymatic defense system. When plants are under stress, the activity of the enzymatic defense system of plant cells is inhibited. Plants contain antioxidant enzyme systems such as superoxide dismutase (SOD), peroxidase (POD), and catalase (CAT), which can remove intracellular reactive oxygen species (ROS) and free radicals. These antioxidant enzyme systems are of great significance to maintain the balance of ROS metabolism in plants and stabilize the cell membrane stability index (CMSI) in plants. When plants are under stress, SOD can catalyze the dismutation of free radicals in plant cells into H_2_O_2_ and O_2_^−^, and POD and CAT also participate in the elimination of H_2_O_2_ [26]. The timely scavenging of free radicals and ROS greatly improves the resistance ability of plants under stress.

In this study, we observed the performance characteristics of potato plants treated with normal light, weak light, and exogenous Ca^2+^, to judge the influence of Ca^2+^ and weak light on potato plants. The objectives of this study were to analyze the physiological characteristics and response genes of potato plants under weak light and exogenous Ca^2+^ treatment. These results can help further clarify the mechanism of Ca^2+^ in regulating plant growth under weak light. 

## 2. Results

### 2.1. Effects of Exogenous Ca^2+^ Treatment Morphological Indexes under Weak Light of Potato

**Figure 1:**
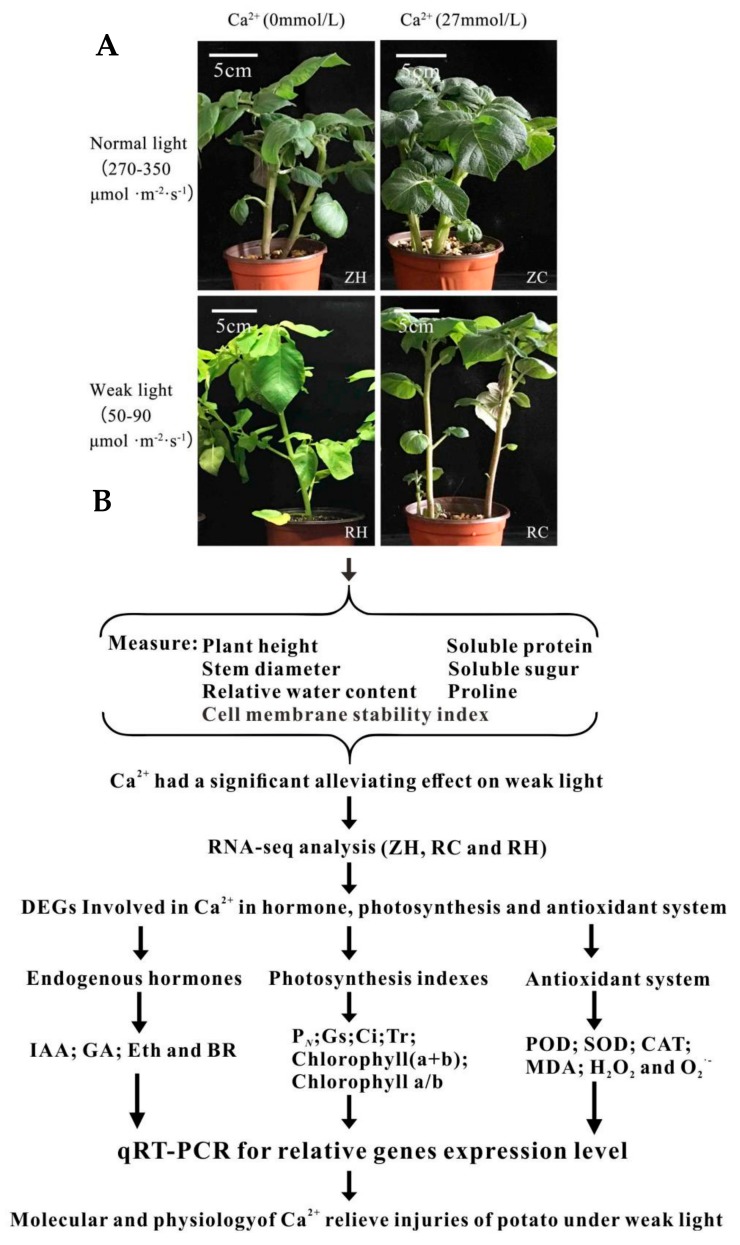
Potatoes were photographed and observed after treatment. (**A**) Potatoes served as a control group under normal light (ZH); the state of exogenous Ca^2+^ under normal light (ZC); potatoes group under weak light (RH); the state of exogenous Ca^2+^ under weak light (RC). (**B**) experimental design and flow chart designed for hormone, photosynthesis, antioxidant, and transcriptome analyses.

**Figure 2:**
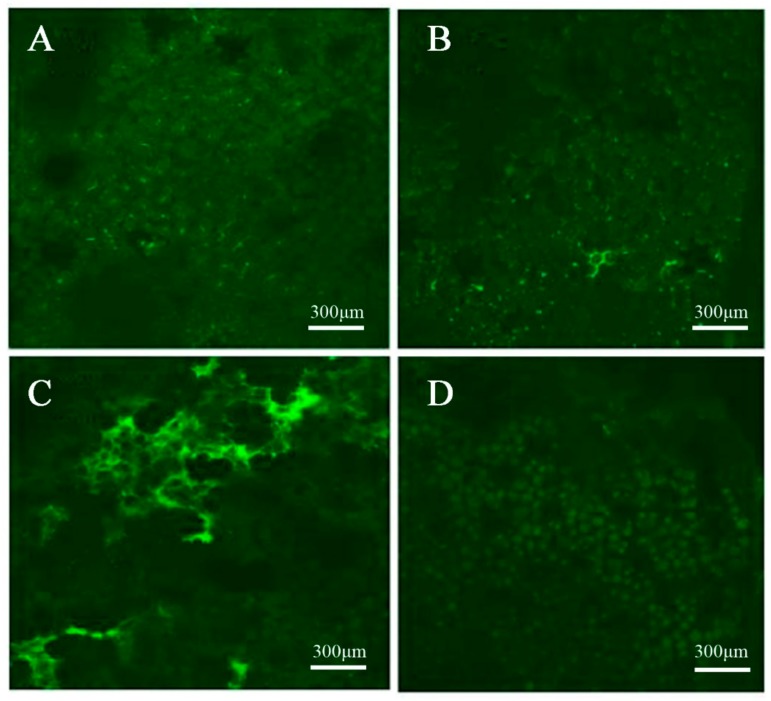
Ca^2+^ fluorescence signals localized in different treatment groups. (**A**) The control group did not contain any treatment (ZH). (**B**) Exogenous Ca^2+^ was treated under normal light (ZC). (**C**) Ca^2+^ distribution in potatoes under weak light (RH). (**D**) Effects of exogenous Ca^2+^ treatment on the distribution of endogenous Ca^2+^ in potatoes under weak light (RC).

In our study, potato seedlings were obtained from an artificial climate box after treatment with weak light (RH and RC group) and Ca^2+^ (ZC and RC), whereas the CK (Control Check) comprised untreated seedlings (ZH). Photos of the development processes of the different treatments were taken with a camera (Figure 1A). As shown in Figure 1, under the condition of weak light, the potato leaves were yellow, the stems were thinner, and the plants were taller. In contrast, when exogenous Ca^2+^ was sprayed, plants grew vigorously, and physiological characters returned to the normal growth level. Through Ca^2+^ fluorescence signal localization, we can find that Ca^2+^ was evenly distributed in the cell membranes and intercellular spaces of potato leaves under normal light (Figure 2). After weak light treatment, the distribution of Ca^2+^ changed, and the fluorescence signal of some cells was significantly enhanced, indicating that a weak light signal induced the flow of Ca^2+^ between cells. After applying exogenous Ca^2+^ to leaves, the fluorescence intensity of Ca^2+^ in each cell did not change significantly under normal light. We measured the height (H), stem diameter (SD), and relative water content (RWC) under different treatments. H was significantly higher in RH compared to ZH under weak light, while RH stems were thinner than those of ZH (Table 1); these morphological changes may lead to a potential risk of lodging. When the RH plants were treated with exogenous Ca^2+^, H and SD of the RC plants all recovered to the level of ZH. The RWC of RC was higher than RH, while there were no significant differences in other groups (Table 1), indicating that exogenous Ca^2+^ can affect RWC under weak light treatment. We suspect that RWC is a relatively stable trait and not sensitive to weak light stress. Overall, Ca^2+^ treatment changed the morphology of potato plants, reducing the abnormal growth of the plant under weak light stress.

### 2.2. Physiological Indexes of Exogenous Ca^2+^ Related to Homeostasis 

**Figure 3:**
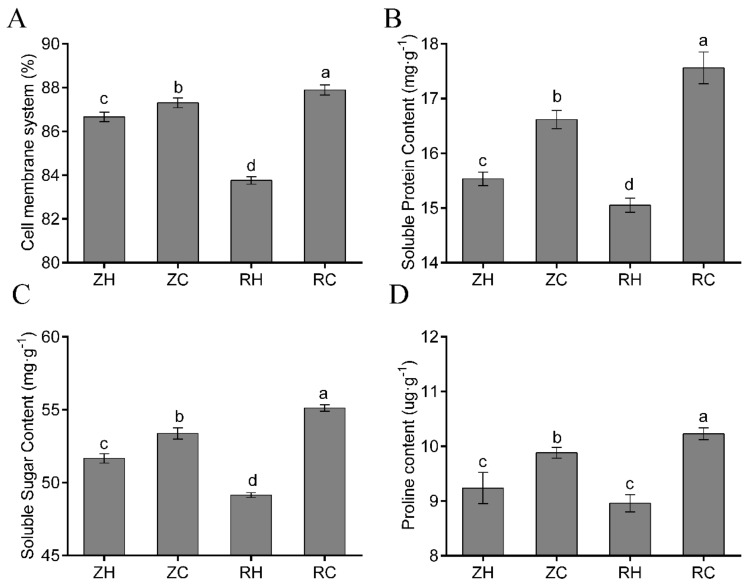
We determined the physiological indexes of Ca^2+^ regulation in weak light. (**A**) Cell membrane system, (**B**) superoxide anion and soluble protein, (**C**) soluble sugar, and (**D**) proline were determined. Values represent the mean ± S.E. (*n* = 3). Letters (a, b, c, d) indicate significant differences at *p* < 0.05 according to Duncan’s multiple range tests.

In order to study the effects of exogenous Ca^2+^ to relieve weak light stress on the internal steady-state system, we analyzed the indicators of CMSI, soluble protein (SP), soluble sugar (SS), and proline (PR) in potato plants. CMSI was markedly reduced by weak light treatment. When the plants were sprayed with Ca^2+^, the CMSI significantly increased (Figure 3A). Similarly, the SP and SS contents were significantly affected by weak light and exogenous Ca^2+^; weak light reduced the content, and Ca^2+^ increased the content (Figure 3B,C). The PR content was not affected by weak light; there was no significant difference between ZH and RH plants, but the effect of exogenous Ca^2+^ was significant, as the PR content in RC plant was highest compared to other groups (Figure 3D). Exogenous Ca^2+^ exhibited a stable effect on increasing homeostasis material in plants, and the increasing extents were 14.29%, 10.83%, and 12.41% in each RH group, respectively. 

### 2.3. RNA-seq Assembly of the Potato Transcriptome Reference

To study the differences in RNA-seq between weak light and exogenous Ca^2+^ treatment in potatoes, three biological replicates were performed on nine cDNA samples (ZH1, ZH2, ZH3, RH1, RH2, RH3, RC1, RC2, and RC3). Nine cDNA libraries were sequenced by Illumina HiSeq to detect the transcriptome levels of exogenous Ca^2+^ gene expressions in relieving injuries to potatoes under weak light. High-quality, clean reads were obtained by filtering low-quality reads obtained by sequencing. The Q30 of nine samples exceeded 93.39%, and the base content was uniform (Table 2). An average of 79.30% of reads was aligned to the solanum tuberosum reference genome (http://solanaceae.plantbiology.msu.edu/index.shtml). A total of 13,640 DEGs were identified in RH, RC and ZH. Pearson’s significant correlation (Figure S1) between the fragments per kb per million reads (FPKM) distributions of biological replicates of all samples confirmed the high reproducibility of the sequencing data. Thus, the assembly quality of the transcriptome was satisfactory. The raw RNA-seq data used in this study have been deposited in the National Center for Biotechnology Information (NCBI) Sequence Read Archive (SRA) database under the accession number SRP212030. 

### 2.4. Comparative Analysis of DEGs

**Figure 4:**
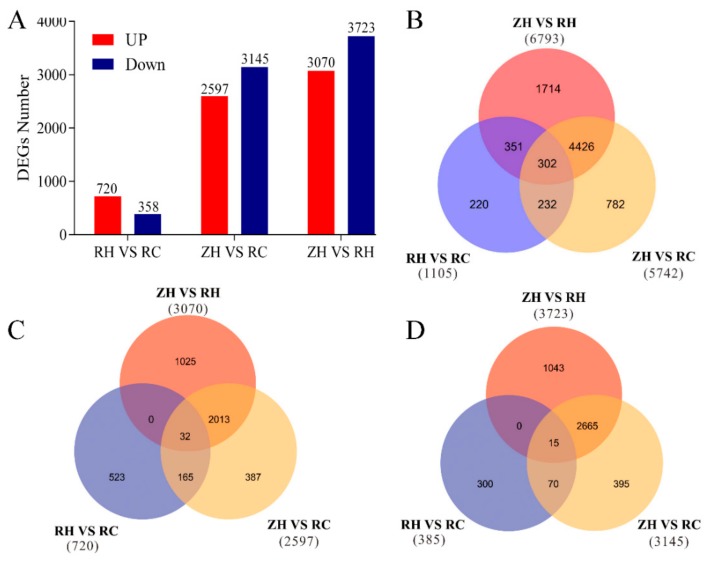
Transcriptome data differential gene numbers and Venn diagram. (**A**) For the RNA-seq database, the number of all genes and the number of up/down-regulated genes in different periods and treatments. Up-regulated numbers are shown in red, while the number of down-regulated genes is shown in blue. (**B**) Venn diagram of the DEGs in different comparisons. The numbers indicate unique and common DEGs in three replicates for different comparisons. (**C**) Venn diagram of the up-regulated genes in different comparisons. (**D**) Venn diagram of the down-regulated genes in different comparisons.

DEGs were analyzed using the FPKM method to determine the degree of overlap between the three comparison groups (Figure 4). Compared with the potato, the total numbers of up-regulated genes in potatoes treated with weak light and Ca^2+^ were 3070 and 720, respectively. The numbers of down regulated genes in the ZH vs. RH and RH vs. RC groups were 3723 and 358, respectively. Compared with the RC group, the RH group contained 523 up-regulated genes and 300 down-regulated genes (Figure 4C,D). Various process genes were compared using Venn diagrams. A total of 1078 DEGs were obtained in the RH vs. RC group, 5742 DEGs were obtained in the ZH vs. RC group, and 6793 DEGs were obtained in the ZH vs. RH group. A total of 302 DEGs (32 commonly up-regulated and 15 commonly down-regulated) were shared among the three treatments (Figure 4B–D); thus, implying that these 302 DEGs might be responsible for relieving injuries to potatoes under weak light. A correlation heat map analysis of the expressions among samples revealed reproducible results (Figure S1). A Venn diagram was made based on common DEGs, in Figure 4C,D, suggesting that exogenous Ca^2+^ affected the expression of related genes of potatoes under weak light.

### 2.5. GO and KEGG Enrichment of Potatoes under Weak Light and Exogenous Ca^2+^ Treatment

**Figure 5:**
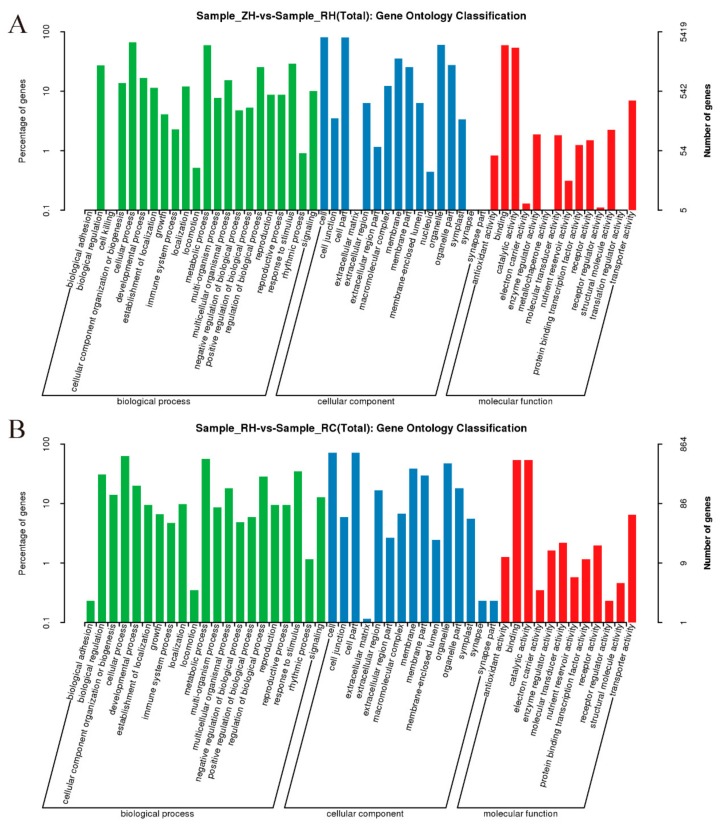
Scatter plot of the GO terms for the DEGs. (**A**) GO analysis of ZH vs. RH. ‘Percentage of gene’ is the ratio of the DEGs to the background number in the given GO classification. Go classification of DEGs enriched under weak light. (**B**) GO annotation classification of potatoes with weak light by exogenous Ca^2+^.

To determine the similarities and differences in potato transcriptomes between weak light and exogenous Ca^2+^, DEGs were used to perform GO classification and KEGG functional enrichment analyses. The GO terms in ZH vs. RH and RH vs. RC were assigned to three categories: Biological process (BP), cellular component (CC), and molecular function (MF) (Figure 5A,B). For the DEGs in ZH vs. RH, significant GO terms were mostly enriched in the cellular process, metabolic process, and in response to stimuli in the BP category. Most of the DEGs in the CC category were assigned to the organelle, cell parts, and the cell. The top three GO terms in the MF category were transporter activity, catalytic activity, and binding (Figure 5A). For the DEGs in RH vs. RC, only cellular process, metabolic process, and response to stimuli terms were significant in the BP category; cell, cell part, and organelle terms in the CC category; and binding and catalytic activity terms in the MF category (Figure 5B). The most frequent GO terms were binding, cell, and cellular process (Figure 5). Therefore, the corresponding genes of these significant terms might play important roles in exogenous Ca^2+^ to relieve injuries to potatoes under weak light.

**Figure 6:**
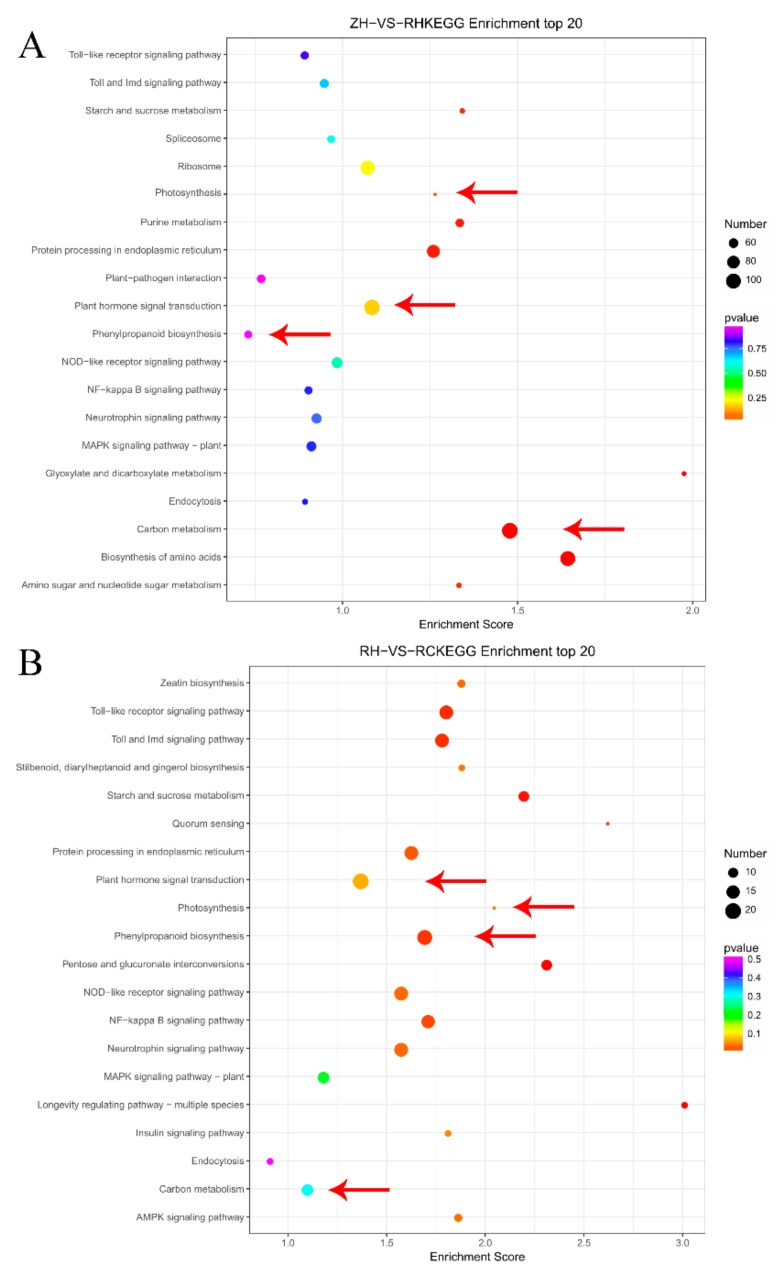
The top 20 enriched KEGG pathways were analyzed for the DEGs. ‘Enrichment factor’ is the ratio of the DEGs to the background number in the given pathway. The dot size represents the number of genes, and the color represents the q-value range. (**A**) The top 20 KEGG pathways were analyzed in ZH vs. RH. (**B**) The top 20 KEGG pathways were analyzed in RH vs. RC.

The top 20 KEGG enrichment pathways in two comparisons are represented in Figure 6. DEGs were largely enriched in carbon metabolism, photosynthesis, phenylpropanoid biosynthesis, and plant hormone signal transduction pathways in ZH vs. RH (Figure 6A). Similarly, plant hormone signal transduction was also enriched in RH vs. RC. In photosynthesis (Ko00195), 39 and nine DEGs were detected, and seven genes were co-expressed in the ZH vs. RH and RH vs. RC groups, respectively. In plant hormone signal transduction (Ko04075), 106 and 20 DEGs were detected in the ZH vs. RH and RH vs. RC groups, respectively. Among the 20 DEGs compared in RH vs. RC (Figure 6B), eight DEGs were assigned to auxin, three DEGs were assigned to cytokinin, one DEG was assigned to abscisic acid, three DEGs were assigned to ethylene, and three DEGs were assigned to salicylic acid. The different expression hormones and photosynthesis DEGs between weak light and exogenous Ca^2+^ suggest that plant hormones and photosynthesis play a key role in relieving injuries to potatoes under weak light. 

### 2.6. Overview and Analysis of Regulatory Pathways of DEGs

**Figure 7:**
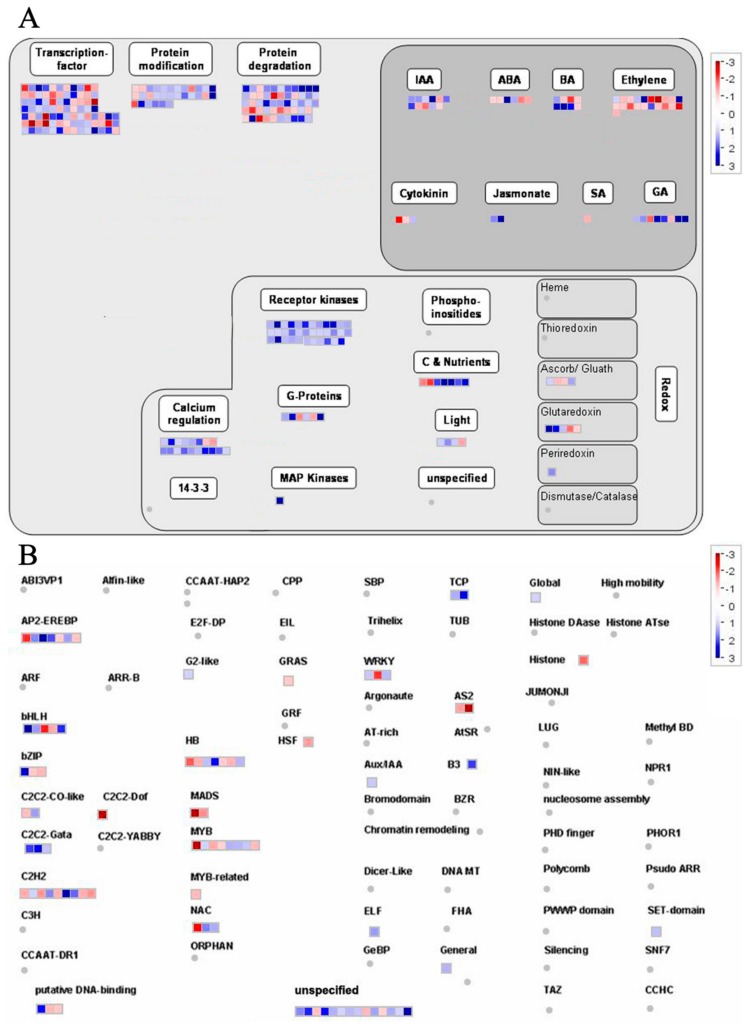
Overview of gene expression and transcription responses visualized using Mapman software in RH vs. RC. (**A**) Overview of regulation enrichment among DEGs in comparing RH and RC under weak light. (**B**) Main transcription factor (TF) family enrichment pathway among DEGs. The transcriptional changes in weak light and exogenous Ca^2+^. Genes in blue and red indicate up-regulation and down-regulation in RH and RC samples relative to DEGs. Individual genes are represented by small squares. The scale bar displays transformed log_2_ fold changes.

Regulation overview pathways in RH vs. RC were analyzed using MapMan (Figure 7A, Table S1), and the majority of DEGs were up-regulated and functionally enriched in TFs (transcription factors), including MYB, C2H2, bHLH and AP2/EREBP (Figure 7B, Table S1B). Other pathways including calcium regulation, receptor kinases, C and nutrients and light, were also up-regulated or down-regulated in RH vs. RC in response to relieving potato injuries under weak light (Figure 7A). Most DEGs were linked to hormones associated with up-regulation of IAA, GA, BA, ABA, ethylene, and Jasmonic acid (Figure 7A, Table S1A). TFs are major regulators of gene expression and perform significant functions in the transcriptional reprogramming of exogenous Ca^2+^ to relieve injuries to potatoes under weak light. We displayed the expressions of putative TFs that could be classified into 42 TF families in RH vs. RC (Figure 7B). Highly expressed TFs, including PGSC0003DMG400006394 and PGSC0003DMG400022759, were identified in RH vs. RC. PGSC0003DMG400006394 belongs to the bHLH (Basic helix-loop-helix) TF family and the bHLH family of proteins is a group of functionally diverse transcription factors found in both plants, while PGSC0003DMG400022759 is annotated to encode the heat stress transcription factor AP2/EREBP (APETALA2 and ethylene-responsive element binding proteins). These TFs seem to be connected with abiotic or biotic stress responses according to annotation information. Low-expression TFs with fluctuating performances were excluded from further analyses.

### 2.7. Changes in Endogenous Hormone Content and Related DEGs in Potatoes

**Figure 8:**
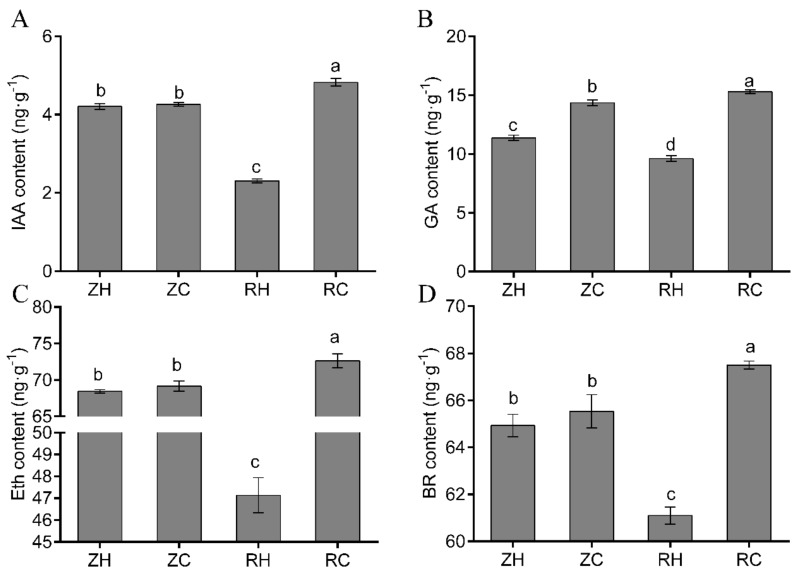
Effects of exogenous Ca^2+^ and weak light on endogenous hormone in potatoes. We measured the levels of (**A**) IAA, (**B**) GA, (**C**) Bra, and (**D**) Eth in different treatments. Values represent the mean ± S.E. (*n* = 3). Letters indicate significant differences at *p* < 0.05 according to Duncan’s multiple range tests.

Contents of endogenous IAA, Eth, GA and BR in weak light presented a quickly declining trend, with the plant growing weaker, and IAA, Eth, GA, and BR contents of weak light stress reached the lowest level in the RH group (Figure 8). After Ca^2+^ treatment, endogenous IAA, Eth, GA, and BR contents of the RC group were dramatically increased with respect to the ZH (CK) group, whereas Ca^2+^ treatments in the ZC group saw no significant difference (Figure 8). We speculate that the Ca^2+^ treatment could stimulate the accumulation of some hormones under weak light, so as to quickly adapt to the changes of the weak light environment and support growth and development of the plants. In the RH vs. RC group, we found that most of the genes related to plant hormone metabolism were down-regulated. There were 17 ETH-related genes in total, 13 of which were down-regulated, among which the down-regulated expressions of PGSC0003DMG400013255 and PGSC0003DMG400010285 were 8.05-fold and 5.10-fold (Table S1). Six genes were identified related to BR, three of which were down-regulated. In addition, nine genes were related to IAA, five of which were down-regulated (Table S1). 

### 2.8. Effects of Exogenous Ca^2+^ on Chlorophyll Content and Photosynthesis under Weak Light

**Figure 9:**
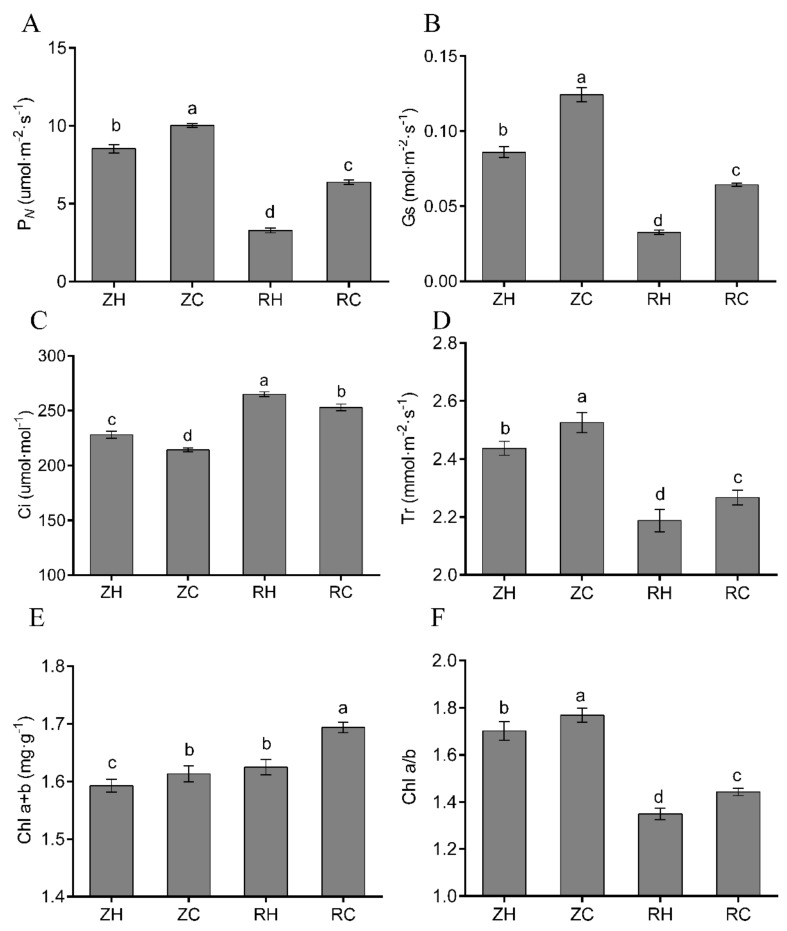
Effects of exogenous Ca^2+^ and weak light on photosynthesis and chlorophyll content in potatoes. We measured the levels of (**A**) P*_N_*, (**B**) Gs, (**C**) Ci, (**D**) Tr, (**E**) chlorophyll and (**F**) chlorophyll a/b in different treatments. Values represent the mean ± S.E. (*n* = 3). Letters indicate significant differences at *p* < 0.05 according to Duncan’s multiple range tests.

Chloroplasts are the main site of photosynthesis, and the number of and damage to of chloroplasts directly affect photosynthesis. Chl a and b are the main indicators for measuring chloroplasts. The changes in Chl (a + b) content showed a gradual upward trend in response to weak light and exogenous Ca^2+^ treatment (Figure 9E). Relative to the normal light group (ZH and ZC), Chl a/b were significantly decreased in the weak light group (RH and RC). However, exogenous Ca^2+^ increased Chl (a + b) content and Chl a/b. The photosynthesis parameters presented different changes to trends in all treatments (Figure 9A–D). Under weak light stress (RH group), there was a significant decrease in P*_N_*, Gs, and Tr by 61.49%, 59.76% and 10.22% compared to the ZH group, respectively, whereas Ci (Intercellular CO_2_ concentration) was increased by 13.93% in Ca^2+^-treated plants. Ca^2+^ treatment also resulted in the opposite trend in RH and RC groups, which induced increases in P*_N_*, Gs, and Tr; these parameters were reduced by 48.53%, 48.44% and 3.49%, respectively, in Ca^2+^-treated plants compared to the RH group under weak light, whereas Ci was decreased by 4.54% in Ca^2+^-treated plants. RNA-seq analysis showed that seven genes were co-expressed in the RH vs. RC and ZH vs. RH groups. Interestingly, we found that the RH vs. RC group was up-regulated, while the ZH vs. RH group was down-regulated. Notably, the PGSC0003DMG400002312 gene was up-regulated by 23.05-fold, while in the RH vs. RC group, it was down-regulated by 3.26-fold (Table S1). 

### 2.9. Effects of Exogenous Ca^2+^ on the Antioxidant System under Weak Light

**Figure 10:**
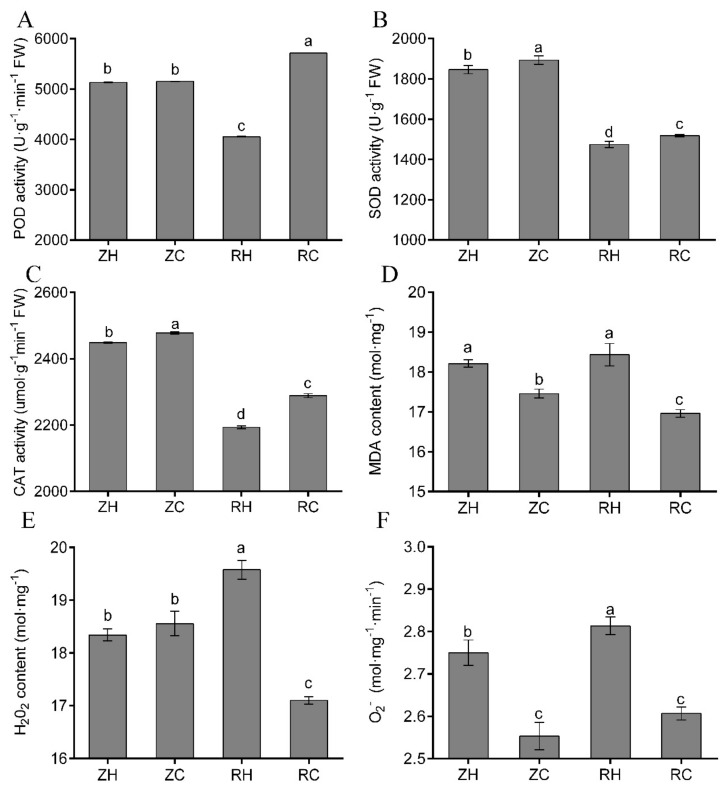
Effects of exogenous Ca^2+^ on the antioxidant system in potatoes. We measured the levels of (**A**) POD activity, (**B**) SOD activity, (**C**) CAT activity, (**D**) MDA content, (**E**) H_2_O_2_ content and (**F**) O_2_^−^ in different treatments. Values represent the mean ± S.E. (*n* = 3). Letters indicate significant differences at *p* < 0.05 according to Duncan’s multiple range tests.

Changes in SOD, POD and CAT activities were similar in the ZH vs RH group, which decreased rapidly response to weak light. However, SOD, POD and CAT activities of RC plants were significantly increased when treated with Ca^2+^ (Figure 10A–C). The MDA content in RH and ZH groups was similar, but when sprayed with exogenous Ca^2+^, MDA content in both ZC and RC decreased (Figure 10D). In normal light conditions, exogenous Ca^2+^ did not change the content of H_2_O_2_, but weak light treatment enhanced the accumulation of H_2_O_2_, and exogenous Ca^2+^ significantly reduced the content of H_2_O_2_ (Figure 10E). Weak light stress increased the rate of O_2_^−^ formation in ZH vs. RH (Figure 10F), and it was decreased in ZC and RC groups. In the transcriptome, we also found that after exogenous Ca^2+^ treatment, the expression of Ca^2+^ regulatory genes were up-regulated up to 5.29-fold (PGSC0003DMG400005063). Genes of PGSC0003DMG400026778 and PGSC0003DMG400005725 related to shade avoidance were down-regulated, and three of the five far-red light related genes were down-regulated. The changes were most obvious in the activated transcription factor bHLH, where three of the five genes were up-regulated and two were down-regulated.

### 2.10. Relative DEGs Measured by qRT-PCR

**Figure 11:**
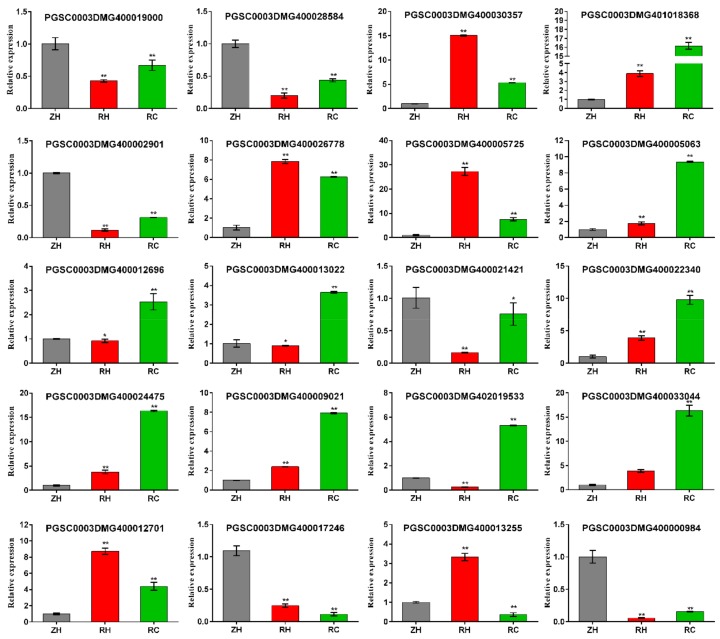
Expression analysis of DEGs under weak light and exogenous Ca^2+^ by qRT-PCR. The experiment included three biological replicates and used SPSS analysis. We chose 20 DEGs including 2 (light), 3 (far-red light), 2 (shade avoidance), 5 (calcium regulation), 3 (GA), 2 (Eth), 1 (BR) and 2 (IAA) (Table S2). Values represent the mean ± S.E. (*n* = 3). Letters indicate significant differences at *p* < 0.05 according to Duncan’s multiple range tests.

**Figure 12:**
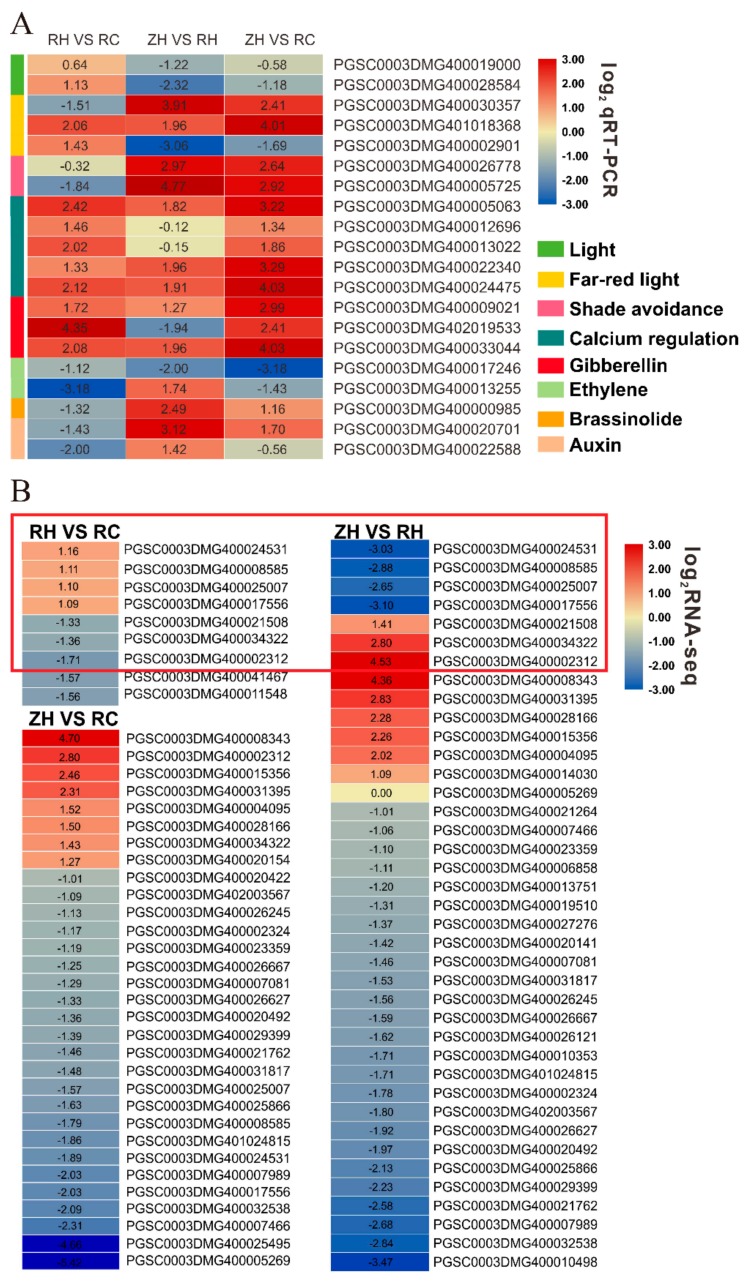
DEGs analyzed in photosynthesis and plant hormones using a heatmap. (**A**) We measured the levels of hormones, light, shade avoidance and calcium regulation in different treatments by qRT-PCR. (**B**) Photosynthesis-related genes were analyzed through the transcriptome in RH vs. RC and ZH vs. RH. The difference in red indicates that both expressed DEGs. Values represent the mean ± S.E. (*n* = 3).

A set of 20 DEGs (Figure 11) were selected for quantitative real-time PCR analysis to confirm their Ca^2+^ response functions under weak light. Comparisons of transcriptome data with qRT-PCR results showed a relatively high correlation (R^2^ = 0.9428) (Figure S2), which verified an accountable RNA-seq analysis in the present research. We chose 20 DEGs including 2 (light), 3 (far-red light), 2 (shade avoidance), 5 (calcium regulation), 3 (GA), 2 (Eth), 1 (BR) and 2 (IAA) (Table S1). Among 20 DEGs, up-regulation of 12 DEGs and down-regulation of eight was confirmed by qPCR in RH vs. RC in accordance with results of the RNA-seq analysis (Figure 11 and Figure 12A).

## 3. Discussion

**Figure 13:**
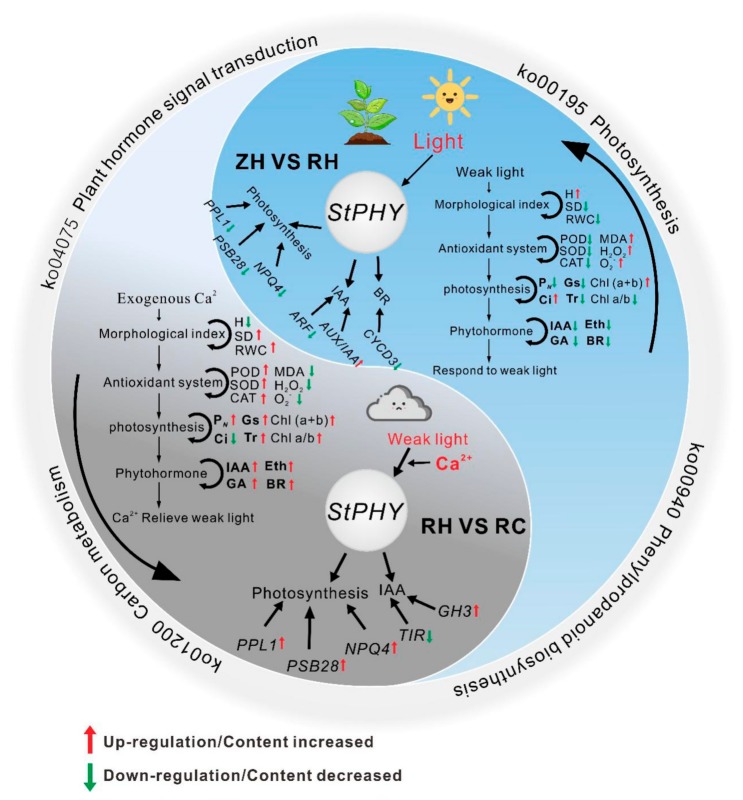
A putative model of a potato plant’s response to weak light and exogenous Ca^2+^. PHY responds to the light signal of the environment, induces a series of actions (ZH vs. RH) in weak light; exogenous Ca^2+^ interfered in the light signal pathway, induces some opposite actions (RH vs. RC) and relieves partial injuries to the potato under weak light. Italics represent genes, the red arrows indicate up-regulation or increased content, and the green arrows indicate down-regulation or decreased content.

Light is an important factor in plant growth and development, which affects almost all growth and development stages of plants. Under weak light conditions, plants can adapt to a weak light environment by changing their morphological structure, physiological and biochemical characteristics, etc., to compensate the adverse effects on plant growth and metabolism [27]. Many studies have shown that the size and morphological structure of individual plants are affected by weak light [28]. Weak light treatment will reduce the dry weight and total dry mass of plants to different degrees. Weak light treatment will not only cause changes in plant individual sizes, but it will also cause the reconstruction of plant morphology. These adaptive changes may help the plants complete generational development, but they are usually harmful to the yield and quality in artificial cultivation. As a large number of elements is needed for plant growth, Ca^2+^ participates in various intracellular metabolism pathways, either directly or as a signal. In our study, we used exogenous Ca^2+^ treatment in potatoes under different light intensities, and we analyzed the related physiological and gene expression changes. Results showed that exogenous Ca^2+^ could significantly relieve injuries to potatoes under weak light stress (Figure 1), which has an important research value and significance for future improvement of potato yields in areas with insufficient light. Based on previous research and our physiological and transcriptomic results, we constructed a model of the abnormal growth of a potato plant regulated by Ca^2+^ under sustained weak light (Figure 13).

### 3.1. RNA-seq Analysis Reveal DEGs under Weak Light 

In this study, we described the gene expression profile of potato responses to weak light and exogenous Ca^2+^. A total of 13,640 DEGs were identified, among which 6793 DEGs were involved with weak light in ZH vs. RH. Moreover, 45.19% of DEGs (3070/6793) were up-regulated and 54.87% (3727/6793) were down-regulated in ZH vs. RH (Figure 4). A total of 1078 different genes were identified when we sprayed exogenous Ca^2+^, among which 720 had up-regulated expressions in RH vs. RC. Moreover, a comparative transcriptome analysis indicated that 97 key genes related to shade avoidance, hormone metabolism and calcium regulation were differently transcribed in the RH vs. RC group (Table S1). The abundant expression of differentially expressed genes may play a significant role in the injuries to potatoes in response to different light intensities [29]. GO and KEGG enrichment analyses indicated that most DEGs were classified in response to weak light stress, including plant hormone signal transduction, phenylpropanoid biosynthesis and carbon metabolism (Figure 4 and Figure 5). We identified a large number of hormone-related genes, and we found that a large number of hormone-related genes changed after exogenous Ca^2+^ treatment. The transcriptome data suggested that a large number of DEGs were involved in various metabolic pathways, most of which were related to the regulation of gene expression, followed by energy production, and metastasis (Figure 5 and Figure 6, Table S1). Based on these comparative transcriptome results, a large number of genes related to calcium regulation, shade avoidance, and hormone metabolism can be verified. Further research should focus on the molecular mechanisms of these genes to better understand the different regulation mechanisms of Ca^2+^-related DEGs in conferring responses to weak light stress.

### 3.2. Effects of Exogenous Ca^2+^ Treatment on Endogenous IAA, GA, BR, and Eth Contents under Weak Light

Endogenous hormones play a significant role during the growth and development of potatoes. Although there are very few endogenous hormones, they are easily affected by the internal environment and regulate the whole process of potato growth. Leaves are the main organ in plant photosynthesis and the metabolic source of plant growth and development [30]. Plant endogenous hormones can affect the growth and development of leaves to a large extent, and the endogenous hormones in leaves are also sensitive to the environment. In our study, the level of endogenous IAA, GA, ETH and BR in potato leaves changed significantly when treated with weak light (Figure 8), and these changes could be recovered, to some degree, after application of exogenous Ca^2+^, which is consistent with the results of Elliott [31]. In this study, exogenous Ca^2+^ could promote the growth of potatoes and stimulate the deficiency of hormone content after weak light stress. The increase of endogenous IAA and GA contents provides the growth demand for plants [32]. BR can increase stress tolerance, delay leaf senescence, enhance Chl synthesis, improve photosynthesis and promote leaf color deepening and turning green [33]. After exogenous Ca^2+^ treatment, BR in potato plants is significantly increased, enhancing the tolerance to weak light stress and inhibiting leaves turning yellow. A certain process of plant growth and development will not be affected by only one hormone; it is usually the result of co-regulation by multiple hormones. In this study, weak light treatment could significantly decrease the content of four hormones compared with normal light (Figure 8), but the hormone content showed an increasing trend after exogenous Ca^2+^ application. However, the content of these four hormones did not change significantly after application of Ca^2+^ to the plants under normal light. These results indicated that exogenous Ca^2+^ could inhibit the degree of abnormal growth of potato plants under weak light, which was achieved by regulating the content of IAA, GA, BR and Eth. Therefore, we speculate that exogenous Ca^2+^ could stimulate the expression of plant hormone-related genes under weak light, and stimulate the increase of related hormone content, so as to relieve weak light stress.

### 3.3. Exogenous Ca^2+^ Relieves The Changes in Physiological Indexes under Weak Light Stress

PR and SS are important osmotic adjustment substances, and the abundance of these two substances is positively correlated with stress resistance [34]. Moreover, MDA and relative electrical conductivity are indicators of lipid peroxidation, and increasing MDA, O_2_^−^ and H_2_O_2_ content conductivity suggest that the membrane systems may be severely damaged [35]. Under normal circumstances, the production and clearance of free radicals in plant cells are in a dynamic balance, and a stable level of ROS is maintained in plant cells to maintain normal cell function and signal transduction. When plants are subjected external stress, this balance is destroyed and the cell membrane is damaged. A large amount of ROS accumulates, causing membrane lipid peroxidation, which can seriously damage plant cells and affect cell metabolism and gene expression [36,37]. To avoid oxidative damage, plants have evolved a series of reactive oxygen scavenging mechanisms, including the water cycle, the ascorbic acid–glutathione cycle, and peroxidase and catalase enzymes [38]. The results showed that the contents of MDA, H_2_O_2_ and O_2_^−^ in potato plants increased significantly, and the stability of the membrane system decreased in weak light (Figure 10). When we used exogenous Ca^2+^, the content of three substances reduced, suggesting that Ca^2+^ can stimulate a stable membrane state in the plant. The accumulation of compatible osmotic-regulating substances is an important physiological mechanism for plants to resist adversity. Under adverse conditions, SS, SP, and PR in plants play important roles in osmotic regulation and cell membrane structure stability [39]. SS and SP can maintain plant leaf turgor and ensure the effective intake of CO_2_ in stomatal conductance. The accumulation of PR is beneficial to the plant to maintain the water content of the leaves and reduce the loss of water caused by the plants under stress. In addition, it has been reported that SS and PR reduce oxidative damage by activating specific ROS scavenging systems and reducing free radical production. This study shows that SS, SP and PR contents all had different degrees of reduction in RC (Figure 3). Results showed that the CMSI decreased after exogenous Ca^2+^ was sprayed; the content of SP, SS and PR increased; and the regulating ability was restored. Ca^2+^ fluorescence signal photos showed that exogenous Ca^2+^ treatment could influence the distribution of endogenous Ca^2+^ in the leaves, then affect the signal transmission, inducing a series of reactions. 

### 3.4. Exogenous Ca^2+^ Relieves The Changes in Photosynthesis and The Antioxidant System under Weak Light Stress

In our study, different treatments had substantially decreased Chl (a+b) contents in weak light. Chl a/b contents were only decreased in exogenous Ca^2+^ (Figure 9E,F). It was reported that decreased Chl content resulted from lipid peroxidation of chloroplasts and thylakoid membranes under heat stress [40]. Weak light stress was shown to cause various changes in Chl and then affect the photosynthesis of plants, and the photosynthetic parameters significantly changed (e.g., altered structural organization of thylakoids, loss of grana stacking, and grana swelling) [35]. Several studies have shown that photosynthesis is hypersensitive to weak light, and it is often the first cellular function to be impaired by weak light stress [41,42]; the results of our study were in accordance with these observations. In this study, the significant reductions in P*_N_*, Gs and Tr in weak light coincided with a markable increase in Ci, indicating that the reduction in photosynthesis was due to non-stomatal limitations (Figure 9A–D). However, the respective increases of P*_N_*, Gs and Tr in exogenous Ca^2+^ (Figure 9A,B,D) likely functioned as a light acclimation mechanism in leaves to prevent metabolic stress [43]. Wang [44] indicated that epibrassinolide could induce the enhancement of plant adaptive responses to weak light stress. Our results demonstrated that weak light stress mediated decline of P*_N_* was also exogenous and Ca^2+^-dependent. Exogenous Ca^2+^ can significantly improve the performance of P*_N_*, Gs, and Tr parameters and improve the photosynthetic performance of plants under weak light. The generation of cellular ROS (such as H_2_O_2_ and O_2_^−^) was shown to cause oxidative damage to components of the photochemical apparatus, leading to overreduction of the photosynthetic electron transport chain [45]. MDA content indicates the degree of lipid peroxidation, and MDA content positively correlates with the extent of damage to cell membranes. In our study, there was a substantial accumulation of H_2_O_2_, O_2_^−^ and MDA under weak light stress, with a greater accumulation in weak light (Figure 10D–F). This greater accumulation of toxic substances in the heat-sensitive cultivar led to severe membrane lipid peroxidation. As an important class of enzymatic ROS scavengers, SOD catalyzes the dismutation of O_2_^−^ into H_2_O_2_ and O_2_ and POD and CAT further convert H_2_O_2_ into H_2_O and O_2_, together helping plants prevent oxidative damage under weak light stress conditions [46]. In this study, we found gradual decreases of POD and SOD activities in weak light. However, the content of POD and SOD increased after exogenous Ca^2+^ treatment in RH vs. RC (Figure 10A,B). These indicated that the activities of SOD and POD were enhanced by moderately exogenous Ca^2+^ under weak light. This may partly relate to increases in O_2_^−^ formation rates and H_2_O_2_ contents observed under weak light. In other words, increased O_2_^−^ and H_2_O_2_ contents served as reaction substrates and products, and it stimulated the activities of SOD and POD in exogenous Ca^2+^ treatment. The activity of CAT was increased under Ca^2+^ treatment, which suggested that CAT activity was more sensitive to weak light than that of SOD and POD. Therefore, we found that exogenous Ca^2+^ could improve the enzyme activities of POD, SOD and CAT in potato plants under weak light, preventing oxidation, changes in the contents of MDA, H_2_O_2_ and O_2_, and increases in the contents of P*_N_*, Gs and Tr in the plants. Transcriptome analysis showed that plant hormone signal transduction, photosynthesis, carbon metabolism and phenylpropanoid biosynthesis were significantly enriched in the RH vs. RC group (Figure 6 and Figure 7). Detection of all photosynthetic pathways found that seven genes were co-expressed in the RH vs. RC and ZH vs. RH groups. Interestingly, we found that the RH vs. RC group was up-regulated, while the ZH vs. RH group was down-regulated; in particular, the PGSC0003DMG400002312 gene was up-regulated by 23.05-fold, while in the RH vs. RC group it was down-regulated 3.26-fold (Figure 12B, Table S1). 

### 3.5. Exogenous Ca^2+^ Relieves Injuries to Potatoes under Weak Light Stress

There may be two main ways for PHY to regulate plant weak light reactions (mainly mediated by PHYs) (Figure 13). One is that PHY acts to inhibit or activate the expression of related hormone metabolism genes. Another approach is that PHY activates or inhibits CaM (or other calcium receptor) binding proteins through the calcium messenger system, and it further regulates the expression of photosynthesis genes, finally causing changes in the photosynthetic parameters and affecting the growth and development of plants (Figure 13). Based on a group that recently identified that exogenous Ca^2+^ could relieve damage to plants under weak light stress, this study further expanded on Ca^2+^ regulation, mediated by PHY, in the weak light reaction mechanism. In this study, we found that the contents of MDA, O_2_^−^ and H_2_O_2_ decreased after exogenous Ca^2+^ treatment under weak light, while the CMSI, SP, SS and PR contents increased (Figure 3). The increase in H and decrease in RWC could reduce the consumption of assimilates, thereby keeping the accumulation of dry matter stable under decreased P*_N_* in weak light; thus, these changes were plant-adaptive responses to weak light stress. In contrast, exogenous Ca^2+^ induced the further increase of Chl (a+b), the further decrease of Chl a/b, and the further decrease of Ci under weak light, indicating that Ca^2+^ could induce the enhancement of plant-adaptive response to weak light stress (Figure 9). Overall, these results provide novel insights into the complexity of Ca^2+^ in relieving injuries under weak light from molecular and physiological perspectives. 

## 4. Materials and Methods 

### 4.1. Plant Materials

The commercial potato cv. Zhongshu 20 (Institute of vegetables and flowers, Chinese academy of agricultural sciences) was planted in pots in artificial climate boxes. Each pot of potato seeds was about 50 g, and a set of experiments were repeated for every 5 pots. The matrix was mixed with perlite, vermiculite and vegetative soil in the same proportion. The environmental conditions of the artificial climate boxes—during the day, 18 °C, 12 h; during the night, 15 °C, 12 h—and the light intensity was 270–350 μmol m^−2^·s^−1^. Seedlings 21 days after emergence with consistent plant growth were selected for weak light and calcium regulation treatments. Selected plants were transferred to an area of light intensity of 50–90 μmol m^−2^·s^−1^ for weak light treatment; the other environmental conditions were kept the same. Then, the weak light treatment plants and the control plants (with a light intensity of 270–350 μmol m^−2^·s^−1^) were sprayed with exogenous Ca^2+^ (CaCl_2_ concentration of 27 mmol/L) every 3 days. Ten days after exogenous Ca^2+^ treatment, three young leaves of each treatment were combined and frozen in liquid nitrogen and stored at −80 °C for analysis. Each experiment was repeated three times, with five plants randomly sampled for subsequent experiments. To simplify the description, we specified three replicates of ZH (Control), RH (Weak light), ZC (Ca^2+^) and RC (Weak light + Ca^2+^) (Figure 1).

### 4.2. Determination of Exogenous Ca^2+^ Treatment Morphology and Osmotic Adjustment Substances under Weak Light

To determine the plant H, SD and leaf RWC, the H and SD were measured with a Vernier caliper and ruler. After weighing the fresh weight of leaves (FW), they were floated on deionized water in the dark at 4 °C for 6 h, and the fresh weight (TW) was measured with an electronic balance. The dry weight (DW) was subsequently obtained by drying the leaf samples at 105 °C degrees for 30 min and then 75 °C for 48 h until the weight remained constant. The RWC of leaves can be obtained according to the calculation formula: RWC (%) = (FW − DW)/(TW − DW) × 100%. 

The functional leaves in the third segment of the growth point of each plant were selected, avoiding cutting the main vein of the leaf. Each 0.2 g blade was placed in a test tube and 5 mL of deionized water was added into each tube. After being stationary at room temperature for 12 h, the conductivity was measured by a conductivity meter (DDS-11D, INESA, Shanghai, China) to determine electrolyte permeability, denoted as EC1. After boiling the water for 30 min, it was cooled to room temperature, and the electrolyte permeability was measured, denoted as EC2. According to the formula REC (%) = K1/K2 × 100, the electrolyte permeability of each middle tube wafer was calculated. The cell membrane stability index (CMSI) was calculated as MSI = [1−(K1/K2)] × 100%, and each experiment was repeated 5 times. The soluble sugar (SS) content was determined by Li [47]. The soluble protein (SP) content and proline (PR) content were determined by referring to Zou [48]. 

### 4.3. Ca^2+^ Fluorescence Signal Localization

According to an improved method combined with Walczysko [49], the blade was cut to a thickness of about 1 mm and a length of 1.5 cm, avoiding the veins. Fluo-3AM-ester was diluted to 10 mmol·L^−1^ with buffer solution containing HEPES, and 5 μL Pluoronic F127 was added per mL of dye solution. The specific steps were as follows: (1) Place the cut tissue into a 1.5 mL centrifugal tube; (2) rinse with HEPES buffer solution 2 times; (3) incubate with fluo-3 am-ester (10 mmol·L^−1^) for 60 min at 37 °C without light; (4) rinse with HEPES buffer solution 3 times; and (5) put it on a glass slide for observation. The confocal laser excitation wavelength was 488 nm, and the emission wavelength was 526 nm. The fluorescence signal was observed by laser scanning confocal microscope.

### 4.4. Determination of Endogenous IAA, Eth, GA and BRs Contents

The fresh fleshy leaves were analyzed in weak light and Ca^2+^ treatment. The extraction, purification, and determination of endogenous levels of IAA, Eth, GA and BRs by UHPLC were performed as described by Li and Zhou [47,50]. The samples were extracted in cold 80% (*v*/*v*) methanol overnight at 4 °C. The supernatant was collected after centrifugation at 6000 rpm for 15 min. The residue was added into 80% methanol extract, about 10 mL for 12 h and then centrifuged 6000 times to combine the supernatant. PVPP (0.1 g/g) (Crosslinking polyvingypyrrolidone, Solarbio, Beijing, China) adsorption phenolic and pigment were added on a 4 °C table (2000 rpm), and oscillated for 1 h at 80 °C in a cryogenic refrigerator. The completely frozen sample was centrifuged at 4 °C and 10,000 r/min for 15 min in order to remove impurities. The efflux was concentrated to dryness in a rotary evaporator, nitrogen blow dried, and passed through a C18 Sep-Pak cartridge (Waters, MA, USA). The dried extracts were dissolved in 0.5 mL of mobile phase (consisting of 100% [*v*/*v*] methanol) and used for UHPLC analysis. These hormone analyses were conducted on a Thermo Fisher Scientific UHPLC UltiMate 3000 (Thermo Fisher Scientific, MA, USA) equipped with a vacuum degasser, a quandary pump, an autosampler, a thermostated column compartment, and a fluorescence detector. A BETASIL C18 column (Thermo Fisher Scientific) (4.6 × 250 mm, 5 mm) was used, and the detection wavelength for fluorescence detection was Eml ^¼^ 254 nm. Each sample (10 mL) was automatically injected at a flow rate of 1 mL min^−1^. Quantification was made by comparing the peak areas with the known amounts of IAA, CTK, GA and BR.

### 4.5. Determination of Exogenous Ca^2+^ on The Photosynthetic Capacity and Chlorophyll Content under Weak Light

The photosynthetic capacity was analyzed on the third day in the seventh fully expanded leaf from the plant center. The P*_N_*, Gs, Ci and Tr were determined using a portable photo-synthesis meter (Li-6400, Li-Cor, Lincoln, NE, USA) between 9:00−11:00 am. Leaf temperatures were maintained at 25 °C. External CO_2_ concentration remained at 380 ± 10 μmol mol^−1^ under weak light and normal light. The Chl contents were measured as described by Strain and Svec [51], with some modifications. Fresh leaf samples (0.2 g) were obtained from the fragments of the third leaves in each treatment group and incubated for 24 h in the dark at 4 °C in 25 mL of acetone/ethanol/water at 4.5:4.5:1 (*v*/*v*/*v*). After filtration, absorbance values were then recorded at 649 and 665 nm. The Chl concentrations were calculated using the following formulae: Chl a = 13.9 A_665_ − 6.9 A_649_; Chl b = 24.9 A_649_ − 7.3A_665_; and Chl a + b = Chl a + Chl b. 

### 4.6. Determination of Exogenous Ca^2+^ on Activities of Antioxidant Enzymes under Weak Light

SOD activities were assayed based on SOD inhibition of nitro-blue tetrazolium (NBT) photochemical reduction at 560 nm. Each 3 mL reaction mixture contained 50 mM phosphate buffer, 0.1 mM EDTA, 13 m Methionine, 75 μM NBT, 2 μM riboflavin, and 50 μL enzyme fraction. One unit of SOD was defined as the amount of enzyme that inhibited 50% NBT photoreduction. 

POD activities were estimated by measuring changes in absorbance at 470 nm due to guaiacol oxidation. Each reaction mixture contained 50 mM phosphate buffer, 10 mM guaiacol, 5 mM H_2_O_2_, and 0.1 mL of enzyme extract. One unit of enzyme activity was defined as the amount of enzyme needed to raise the A_470_ by 0.01 per min. 

CAT activities were assayed according to Aebi [52], which involved spectrophotometric determination of its ability to catalyze the decomposition of H_2_O_2_ at 240 nm. Each reaction mixture contained 25 mM sodium phosphate buffer, 10 mM H_2_O_2_, and 0.1 mL of enzyme fraction. One unit of CAT activity was defined as the amount of enzyme needed to reduce the A_240_ by 0.1 per min. 

The MDA contents were measured in terms of thiobarbituric acid-reactive substances (TBARS) as described by Heath and Packer [53]. Fresh leaf samples (0.5 g) were homogenized in 5 mL of 10% TCA. The homogenates were centrifuged at 10,000× *g* for 10 min at 4 °C. One milliliter of each supernatant was mixed with 2 mL of 10% TCA containing 0.65% TBA. The mixture was heated at 100 °C for 10 min, quickly cooled to room temperature, and then centrifuged at 10,000×*g* for 10 min at 4 °C. Absorbance values of the supernatants were read at 532 and 600 nm against a reagent blank. MDA contents were calculated using an extinction coefficient of 0.155 mM^−1^cm^−1^. Thiobarbituric acid (TBA) colorimetry was used. 

The O_2_^−^ content was determined by the method of Li [54]. O_2_^−^ reacts with hydroxylamine hydrochloride to form NO_2_^−^. NO_2_^−^, under the action of p-aminobenzene sulfonamide and naphthalene ethylenediamine hydrochloride, reacts to form a red azo compound, with a characteristic absorption peak at 530 nm, and O_2_^−^ content in the sample can be calculated according to the A_530_ value.

H_2_O_2_ content was determined by biochemical methods. Fresh leaf samples (0.5 g) were homogenized in 5 mL of ice-cold acetone and centrifuged at 12,000× *g* for 20 min at 4 °C. One milliliter of each supernatant was mixed with 0.1 mL of 20% titanium reagent and 0.2 mL of ammonium solution. The mixture was then incubated at 25 °C for 10 min, precipitated, and dissolved in 3 mL of 2 M H_2_SO_4_. The absorbance values were determined at 415 nm, and the H_2_O_2_ contents were calculated using a standard H_2_O_2_ curve.

### 4.7. cDNA Library Construction and RNA-seq Analysis

According to the above microscopic observation and analysis, RNA-seq analysis was performed in exogenous Ca^2+^ and shading. To simplify the description, we specified three replicates of ZH (Control), RH (Weak light) and RC (Weak light + Ca^2+^). Total RNA was extracted from 9 samples of exogenous Ca^2+^ and shade samples using the mir-Vana miRNA Isolation Kit (mirVana™ miRNA isolation Kit, Ambion-1561, MA, USA) following the protocol of the manufacturer. The libraries were constructed using TruSeq Stranded mRNA LTSample Prep Kit (Illumina, San Diego, CA, USA) according to the instructions of the manufacturer. Then, these cDNA libraries were sequenced on the Illumina sequencing platform (Illumina HiSeqTM 2500, San Diego, CA, USA), and 125 bp/150 bp paired-end reads were generated. The transcriptome sequencing and raw reads were processed by Trimmomatic [55]. The reads containing poly-N and low-quality reads were removed to get a clean read. The clean reads were then mapped to the reference genome using hisat2 [56].

### 4.8. Analysis of DEGs, Cluster Analysis, GO, and KEGG Enrichment

FPKM values of all genes were calculated by cufflinks, and the read counts for all genes were obtained using htseq-count [57]. For transcriptional-level quantization, the FPKM and read counts (protein coding) values for all transcripts were calculated by bowtie2 and express [58]. DEGs were identified using the DESeq R-packet function estimation size factor and nbinom test. *p* value < 0.05 and fold change >2 or <0.5 were set as the threshold for a significant difference. Hierarchical clustering analysis of DEGs was analyzed to explore gene expression patterns. Based on the hypergeometric distribution, GO enrichment and KEGG pathway enrichment analyses of DEGs were indicated in the R program. The read portion was reassembled by StringTie [59]. The reference genome and known annotated genes were then aligned using cuffcompare software for gene structure extension and new transcript identification. We reference to the potato (solanum tuberosum) genome [60]. (Version: 3.4, http://solanaceae.plantbiology.msu.edu/index.shtml).

### 4.9. Functional Annotation and KEGG Enrichment Pathway Analysis

DEGs are characterized by GO and KEGG enrichment (http://www.genome.jp/kegg/) to characterize biological functions and significantly enrich metabolic pathways or signal transduction pathways. Based on Wallenius’s non-central hyper-geometric distribution [61], DEGs were submitted to GO enrichment analysis by the GO-seq R package for enrichment analysis. The statistical enrichment of DEGs in the KEGG pathway was detected by the KOBAS 3.0 [62] website (http://kobas.cbi.pku.edu.cn). Then, the MapMan tool (https://mapman.gabipd.org) was used to display a graphical overview of the metabolic and regulatory pathways.

### 4.10. qRT-PCR Analysis

To explore the expression patterns of 2 (light), 3 (Far-red light), 2 (Shade avoidance), 5 (Calcium regulation), 3 (GA), 2 (Eth), 1 (BR) and 2 (IAA) samples, analyses were performed in weak light and Ca^2+^ treatment conditions using real-time qPCR according to an improved method. Total RNA samples were isolated using RNA-prep Pure plant kit (Tiangen, DP432, Beijing, China). The DNase-treated RNA was extracted, and cDNA was reverse transcribed [63] by the Prime Script™ RT Reagent Kit (TaKaRa, RR047A, Dalian, China). Quantitative real-time PCR was then implemented with the TB Green™ Premix Ex Taq™ II (TaKaRa, RR820A, Dalian, China). The relative expression level of *Actin* gene was analyzed as an internal reference for data standardization. Three biological replicates were performed for all samples, and the relative expression was analyzed as 2^−ΔΔCt^ [64]. The Real-time qPCR primer sequences are shown in Table S3. The results are displayed as the mean ± SD (*n* = 3 biological replicates). The statistics of the relative expressed data were analyzed using SPSS software. Every sample had three biological replicates. 

### 4.11. Statistical Analysis

The data were statistically analyzed with SAS software using Duncan’s multiple range test at the *p* < 0.05 level of significance. MapMan software was used to analyze DEGs in this study (Version: 3.6.0).

## 5. Conclusions

In our study, we conducted a comparative RNA-seq analysis to explain the molecular mechanisms and DEG expressions of exogenous Ca^2+^ to relieve injuries to potato plants under weak light. We explained the mechanism of exogenous Ca^2+^ to relieve injuries under weak light from molecular and physiological perspectives. In summary, our observations suggest that the application of exogenous Ca^2+^ increased CMSI and SP, SS, and PR contents, and it decreased the contents of H_2_0_2_ and O_2_^−^. The photosynthetic parameters P*_N_*, Gs and Tr of plants under weak light were significantly increased when treated with exogenous Ca^2+^, and the antioxidant enzyme activities (POD, SOD and CAT) were enhanced too. RNA-seq analysis indicated that a total of 13,640 DEGs were identified. Moreover, a comparative transcriptome analysis indicated that 90 key genes related to far-red light, shade avoidance, hormone metabolism and calcium regulation were differently transcribed in the RH vs. RC group. In addition, the contents of IAA, BR, GA and Eth were significantly increased after treatment with exogenous Ca^2+^. Overall, these results provide novel insights into the complexity of Ca^2+^ to relieve injuries under weak light, and they are helpful for potato cultivation under weak light stress.

## Figures and Tables

**Table 1:** Height (H), stem diameter (SD) and relative water content (RWC) under different treatments. Treatments are ZH (Control), RH (Weak light), ZC (Ca^2+^), and RC (Weak light + Ca^2+^). Letters indicate significant differences at *p* < 0.05 according to Duncan’s multiple range tests.

Sample	ZH	ZC	RH	RC
H	18.94 ± 0.62b	19.17 ± 0.56b	23.42 ± 0.62a	18.06 ± 0.31b
SD	10.72 ± 0.51ab	11.39 ± 0.32a	10.28 ± 0.25b	11.31 ± 0.35a
RWC	83.37 ± 0.90ab	83.59 ± 0.70ab	82.22 ± 0.78b	85.11 ± 0.66a

**Table 2:** Quality assessment of raw RNA-seq data.

Sample	Clean Reads (Million)	Clean Bases (Gigabytes)	Valid Bases (%)	Q30 (%)	GC (%)
RC1	83.61	12.53	97.93%	94.30%	44.00%
RC2	89.95	13.48	97.75%	93.97%	44.00%
RC3	67.45	10.11	96.79%	93.47%	44.00%
RH1	81.78	12.26	97.63%	93.77%	44.00%
RH2	70.08	10.50	97.79%	94.08%	43.50%
RH3	92.74	13.90	97.27%	93.44%	44.50%
ZH1	85.06	12.75	97.88%	94.12%	43.00%
ZH2	83.99	12.59	97.81%	94.06%	43.00%
ZH3	84.96	12.73	97.28%	93.39%	43.00%

## Supplementary Materials

Supplementary materials can be found at https://www.mdpi.com/1422-0067/20/20/5133/s1.

## Abbreviations

RWC	Relative water content
SD	Stem diameter
H	Height
CMSI	Cell membrane stability index
SS	Soluble sugar
SP	Soluble protein
PR	Proline
O_2_^−^	Superoxide anion
Tr	Transpiration rate
PIF	Phytochrome interaction factor
Ci	Intercellular CO_2_ concentration
Gs	Stomatal conductance
P*_N_*	Net photosynthetic rate
Chl	Chlorophyll
IAA	Auxin
GA	Gibberellin
BR	Brassinolide
MDA	Malondialdehyde
CAT	Catalase
POD	Peroxidase
SOD	superoxide dismutase
CK	Control Check
DEGs	Different expressed genes
BP	Biological process
CC	Cellular component
MF	Molecular function
TF	Transcription factor
GO	Gene ontology
KEGG	Kyoto Encyclopedia of Genes and Genomes
ROS	Reactive oxygen species
FPKM	Fragments per kb per million reads
