# Supplementary material for: Physiological and Transcriptomic Analyses Elucidate That Exogenous Calcium Can Relieve Injuries to Potato Plants (Solanum tuberosum L.) under Weak Light
Source: Int J Mol Sci. 2019 Oct 16;20(20):5133. doi: 10.3390/ijms20205133 (PMC6829426; doi:10.3390/ijms20205133)
Supplement: Supplementary file 1 [file ijms-20-05133-s001.zip › Potato Supplementary-Figure.docx]

**Supplementary**


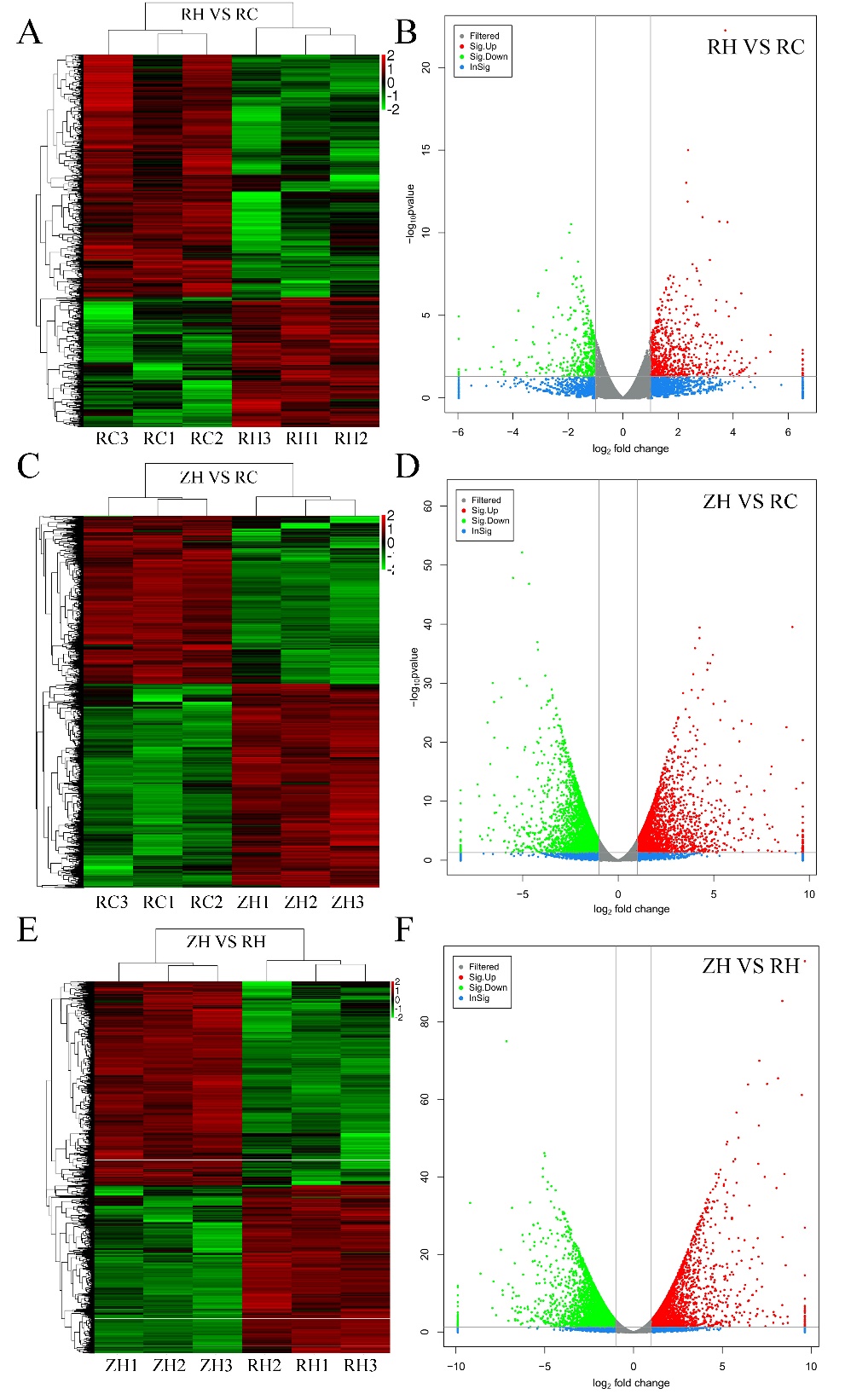


**Fig. S1 All sample heat map and volcano map were analyzed.** Unsupervised hierarchical clustering was performed for differentially expressed genes. The overall distribution of differentially expressed genes can be understood by drawing volcano map.

**Fig. S2** Correlation of expression levels between RNA-seq and qRT-PCR that picked 20 RH VS RC. The log_2_ qRT-PCR (y-axis) was plotted against log_2_ RNA-seq (x-axis) of exogenous Ca^2+^ to relieve injuries of potato under weak light*.*
